# From the archives: Lignin chemistry and vascular cell capacity, chromosome organization in rice meiosis, and circadian clock setting by imbibition

**DOI:** 10.1093/plcell/koad267

**Published:** 2023-10-25

**Authors:** Sophie Hendrix

**Affiliations:** Assistant Features Editor, The Plant Cell, American Society of Plant Biologists; Centre for Environmental Sciences, Hasselt University, Diepenbeek, Belgium

## December 2022: Lignin chemistry and vascular cell capacity

Lignin is a biopolymer in the cell walls of most land plants. Besides its key role in structural support, it is required for water conduction and serves as a physical barrier against pathogens. Lignin is assembled from a wide variety of monomers, and its composition greatly varies among plant species, tissues, and cell types (Vanholme et al. 2019). Ménard et al. (2022) demonstrated the importance of lignin biochemistry in different tracheary element (TE) morphotypes in the plant xylem. Because these cells die before becoming functional, it was long believed that they were unable to adapt to developmental and environmental constraints. However, the authors demonstrated that TEs continue to accumulate lignin for more than 40 days postmortem, resulting in enhanced cell wall stiffness and resistance against negative pressure. All TE morphotypes had specific lignin biochemistries, differing in the amount of lignin, the chemical composition of the lignin units, and their position in longer monomers. Interfering with lignin levels and composition through pharmacologic and genetic approaches resulted in dramatic effects on sap conduction and the ability of plants to recover from drought stress (see Figure). This work showed that postmortem, dynamic fine-tuning of lignin biochemistry is an essential aspect of plant responses to developmental and environmental cues. More recent work of the same research group revealed that the cell type specificity of lignin chemistry is determined by combinations of different laccases (Blaschek et al. 2023).

**Figure.**
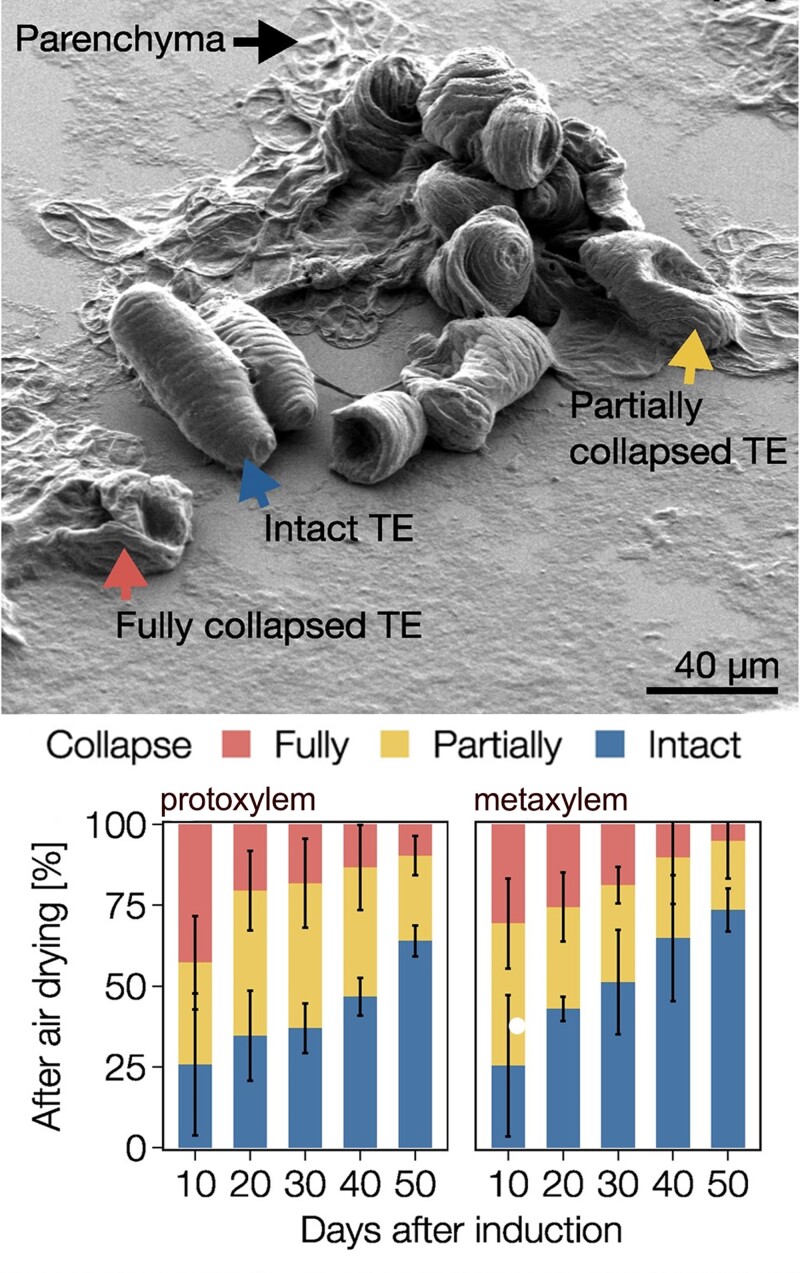
Lignin structure of TEs is fine-tuned during the postmortem maturation of TE morphotypes, enabling dynamic adjustment of their conductive and load-bearing properties to changing developmental and environmental conditions. Parenchymatic cells are collapsed by air drying, which mimics drought by exposing the cells to large water potential differences (scanning electron micrograph, upper panel). During postmortem lignification, there is a gradual increase in resistance to collapse, with the majority of 50-day-old TEs remaining completely intact (lower panel). Adapted from Ménard et al. (2022), Figure 2.

## December 2018: Chromosome organization in rice meiosis

Meiosis is crucial for sexual reproduction in eukaryotes and consists of 1 round of DNA replication followed by 2 rounds of chromosome segregation, resulting in the formation of haploid gametes. The first stage of meiotic prophase I, termed leptotene, is characterized by the gradual loading of cohesin onto the chromosomes to assemble them into thin, thread-structured units. Furthermore, repair of double-stranded DNA breaks (DSBs) is initiated during leptotene, enabling meiotic recombination. In 2018 Zhao et al. (2018) provided evidence for the key role of OsRR24/LEPTOTENE1 (LEPTO1), a type-B response regulator, in leptotene chromosome organization in rice. Response regulators are proteins that regulate cellular responses to environmental cues as part of 2-component signal transduction systems and are involved in a wide variety of cellular responses. The authors revealed that recruitment of meiosis-specific proteins and formation of meiotic DSBs was disturbed in the *lepto1* mutant, resulting in the abolishment of meiotic recombination. Furthermore, they showed that LEPTO1 is required for megasporogenesis and that it likely functions as a transcriptional activator. Yeast 2-hybrid assays demonstrated that LEPTO1 interacts with 2 authentic histidine phosphotransferase (AHP) proteins, which are members of the heat shock protein family. As such, AHP-mediated phosphorylation could be involved in regulating LEPTO1 activity. A year later, another research group confirmed these results in an independent study on another allelic sterile rice mutant of the same gene, which they termed *DEFECTIVE LEPTOTENE CHROMOSOME1* (*DLC1*) (Tao et al. 2019).

## December 1998: Imbibition sets the circadian clock

Rotation of the Earth induces cyclic variation of environmental factors such as light and temperature throughout the day. An endogenous biological clock, also called circadian clock (from the Latin *circa diem*), is found in virtually all kingdoms of life and enables organisms to coordinate their growth, development, and metabolism with these diurnal cycles. In humans, the circadian clock plays essential roles in the regulation of sleep-wake cycles, and its dysregulation is linked to multiple pathologies. In plants, correct functioning of the clock is required to coordinate photosynthesis with light availability, but several other biological processes are also regulated by the clock. This is reflected by the observation that at least 30% of all plant genes show circadian expression patterns (Johansson and Köster 2019). The catalase (CAT) gene family in Arabidopsis is a well-known example. Whereas *CAT1* transcripts do not show rhythmic patterns throughout the day, *CAT2* and *CAT3* mRNA levels peak at dawn and dusk, respectively. In 1998, Zhong et al. (1998) investigated how the circadian clock is initiated in Arabidopsis through kinetic measurements of *CAT2* expression. They found that *CAT2* transcript levels were rapidly induced upon transfer of etiolated seedlings to light and that the amplitude of this induction showed a circadian rhythm, which was synchronized among individual seedlings. Further analyses revealed that the onset and synchronization of the circadian clock were affected by the timing of imbibition but not by the release from stratification. Ten years later, a study by the same research group showed that circadian expression patterns observed in the first 2 days after imbibition depend on a set of 6 clock genes. Furthermore, the authors demonstrated that light is not required for initiation and synchronization of the clock but that it does strengthen the amplitude of the rhythmic patterns observed (Salomé et al. 2008). Not surprisingly, circadian clock regulation is still the topic of many studies today and continues to amaze us “time after time.”

## Dive Curated Terms

The following phenotypic, genotypic, and functional terms are of significance to the work described in this paper:

CAT2 Gramene: AT4G35090
CAT2 Araport: AT4G35090

CAT1 Gramene: AT1G20630
CAT1 Araport: AT1G20630

CAT3 Gramene: AT1G20620
CAT3 Araport: AT1G20620

lignin CHEBI: CHEBI:6457
